# Integrated Analysis of Coding and Non-coding RNAs Reveals the Molecular Mechanism Underlying Salt Stress Response in *Medicago truncatula*

**DOI:** 10.3389/fpls.2022.891361

**Published:** 2022-04-18

**Authors:** Yixin An, Haotian Su, Qichen Niu, Shuxia Yin

**Affiliations:** School of Grassland Science, Beijing Forestry University, Beijing, China

**Keywords:** *Medicago truncatula*, legume, whole-transcriptome RNA sequencing, salt stress, non-coding RNAs (ncRNAs)

## Abstract

Salt stress is among the most severe abiotic stresses in plants worldwide. *Medicago truncatula* is a model plant for legumes and analysis of its response to salt stress is helpful for providing valuable insights into breeding. However, few studies have focused on illustrating the whole-transcriptome molecular mechanism underlying salt stress response in *Medicago truncatula*. Herein, we sampled the leaves of *Medicago truncatula* treated with water or NaCl and analyzed the characteristics of its coding and non-coding RNAs. We identified a total of 4,693 differentially expressed mRNAs (DEmRNAs), 505 DElncRNAs, 21 DEcircRNAs, and 55 DEmiRNAs. Gene ontology and Kyoto Encyclopedia of Genes and Genomes pathway enrichment analyses revealed that their functions were mostly associated with metabolic processes. We classified the lncRNAs and circRNAs into different types and analyzed their genomic distributions. Furthermore, we predicted the interactions between different RNAs based on the competing endogenous RNA (ceRNA) theory and identified multiple correlation networks, including 27 DEmiRNAs, 43 DEmRNAs, 19 lncRNAs, and 5 DEcircRNAs. In addition, we comprehensively analyzed the candidate DEmRNAs and ceRNAs and found that they were involved in Ca^+^ signaling, starch and sucrose biosynthesis, phenylpropanoid and lignin metabolism, auxin and jasmonate biosynthesis, and transduction pathways. Our integrated analyses in salt stress response in *Medicago truncatula* revealed multiple differentially expressed coding and non-coding RNAs, including mRNAs, lncRNAs, circRNAs, and miRNAs, and identified multiple DEmRNA and ceRNA interaction pairs that function in many pathways, providing insights into salt stress response in leguminous plants.

## Introduction

Soil salinity is an increasingly severe global problem that threatens more than 100 countries and approximately 831 million hectares of land (Rengasamy, 2006; Butcher et al., 2016). It is projected that salinization will impact 50% of all arable land by 2050 (Wang et al., 2003; Butcher et al., 2016). The increase in soil salinity will cause ionic, osmotic, secondary, and oxidative stresses in plants and severely limit their growth and productivity (Yang and Guo, 2018a).

To effectively improve the salt tolerance of plants, we need to identify genes and signaling pathways that are important in salt resistance. Over the past two decades, multiple elements of salt tolerance and their regulatory mechanisms have been explored through genetic and biochemical analyses (Yang and Guo, 2018b; Zelm et al., 2020). The salt overly sensitive (SOS) pathway, which comprises SOS1 (Na^+^/H^+^ antiporter), SOS2/CIPK24 (CBL-interacting protein kinase 24), and SOS3/CBL4 (calcineurin B-like protein), represents the best-characterized CBL-CIPK pathway for regulating sodium concentration in the cytosol under salt stress condition (Ji et al., 2013; Manishankar et al., 2018). Different members in mitogen-activated protein kinase (MAPK) cascades function in regulating ionic and reactive oxygen species (ROS) homeostasis by phosphorylating and activating SOS1 and controlling the scavenging of ROS, respectively (Pitzschke et al., 2009; Yu et al., 2010; Pérez-Salamó et al., 2014). In response to osmotic stress induced by salinization, abscisic acid (ABA)-independent sucrose non-fermenting1-related protein kinase 2 (SnRK2s) and ABA-dependent SnRK2s play important roles in transcriptional and post-transcriptional regulation (Kulik et al., 2011; Zelm et al., 2020). In addition, the biosynthesis and transport of auxin and ABA are responsible for salt tolerance (Duan et al., 2013; He et al., 2018; Korver et al., 2018).

With the advancement of next-generation sequencing technologies, whole-transcriptome RNA sequencing has revealed that protein-coding RNAs, as well as non-coding RNAs (ncRNAs), play essential regulatory roles in plants’ response to salt stress (Wang et al., 2017). MicroRNAs (miRNAs) form an extensive class of ncRNA molecules with 20–24 nucleotides; they negatively regulate target mRNA expressions (Zhang, 2015). Previous studies have demonstrated that miRNAs are involved in plants’ response to salt stress. For instance, overexpressing *osa-miR171c* decreased salt stress tolerance in rice (Yang et al., 2017). Also, overexpressing *osa-miR396c* reduced salt stress tolerance in *Arabidopsis thaliana* and rice (Gao et al., 2010). Transgenic *Arabidopsis* overexpressing *miR399f* showed tolerance to salt stress (Baek et al., 2016) and overexpression of *miR156* conferred salt tolerance in alfalfa (Arshad et al., 2017). Long non-coding RNAs (lncRNAs) are defined as a group of ncRNAs with more than 200 nucleotides, which provide an important perspective on the centrality of RNA in gene regulation (Rinn and Chang, 2012). LncRNAs are often grouped into sense, antisense, intronic, bidirectional, and intergenic lncRNAs depending on their location relative to nearby protein-coding genes. They can regulate gene expression *via cis-*acting or *trans-*acting (Ponting et al., 2009). Using whole-transcriptome RNA sequencing, Wang et al. (2015) and Deng et al. (2018) identified and characterized the lncRNAs involved in salt stress in *Medicago truncatula* and *Gossypium hirsutum*. Circular RNAs (circRNAs) are endogenous covalently closed RNAs generated by alternative circularization (Li et al., 2017; Fu et al., 2019). Emerging evidence has demonstrated that plant circRNAs are differentially expressed (DE) under various stress conditions (Li et al., 2017).

Recently, a competing endogenous RNA (ceRNA) theory has been widely accepted as a novel type of gene regulatory model (Salmena et al., 2011). LncRNAs and circRNAs act as ceRNAs to interact with common miRNA response elements and ultimately de-repress the transcriptional or/and translational limitations on miRNA target genes (Ala et al., 2013; Li et al., 2019). Based on this theory, a number of studies have analyzed the ceRNA-miRNA-target gene regulatory networks based on lncRNA/miRNA, circRNA/miRNA, and miRNA/mRNA interactions in plants (Xu et al., 2016; Li et al., 2019; He et al., 2020). However, to the best of our knowledge, the comprehensive studies of the mRNA, miRNA, lncRNA, circRNA, and ceRNA networks in salt stress response in *Medicago truncatula* are lacking. In addition, the functions of these networks have not been extensively clarified.

*Medicago truncatula*, a close relative of alfalfa, is a model plant widely used in the study of legumes due to its simple genomic ploidy, small genome size, and short growth cycle (Tang et al., 2014). Considering the severe trend of soil salinization, there is a need to unravel the molecular mechanisms underlying salt stress responses in *Medicago truncatula* to provide important instructions in breeding practice for legumes. Therefore, in this study, we used whole-transcriptome RNA sequencing to identify and characterize coding and non-coding RNAs, and the ceRNA networks in *Medicago truncatula* treated with NaCl or water. Our results extend the current view on non-coding RNAs as ubiquitous regulators under salt stress and further deepen our understanding of the molecular mechanisms underlying salt stress response in *Medicago truncatula*.

## Materials and Methods

### Plant Growth Conditions and Salt Treatment

Sterilized seeds of Jemalong A17, an ecotype of *Medicago truncatula*, were kept at 4°C for 2 days. After vernalization, the seeds were placed in the Murashige and Skoog medium to germinate at 25°C. Afterward, the sprouted seedlings were transplanted into plastic pots filled with vermiculite and nutrient soil in a ratio of 1:1. The seedlings were cultured at 25°C with a 16 h light/8 h dark cycle and a relative humidity of 75%. After 3 weeks, the control group (CK) was continuously watered with neutral water, whereas the experimental group (Salt) was watered with a solution of 300 mmol/L NaCl. After 1 week, leaves of the CK and Salt groups were collected for whole-transcriptome RNA sequencing (with 3 biological repetitions) in Gene *Denovo* Biotechnology Co., Ltd (Guangzhou, China).

### RNA Extraction and Sequencing

Leaves of the CK and Salt groups were collected (with three biological repetitions) and frozen in liquid nitrogen. Total RNA extraction was performed from 6 samples using TRIzol reagent (Invitrogen). RNA quality was estimated using a NanoDrop 2,000 spectrophotometer (Thermo Fisher Scientific). After ribosomal RNA (rRNA) removal, template RNA fragmentation (200–500 nucleotides), cDNA synthesis, and PCR amplification, we used Illumina HiSeq™ 4000 for total RNA sequencing. The raw reads were first quality-controlled with FASTQ by filtering low-quality reads (Chen et al., 2018). Afterward, clean reads were aligned to the *Medicago truncatula* genome (MedtrA17_4.0) using the HISAT2 software (Kim et al., 2015).

### mRNA Identification and Analysis

Based on the genomic mapping results, we reconstructed transcripts using Stringtie to identify protein-coding genes (Pertea et al., 2015). The fragments per kilobase per million reads (FPKM) values were obtained to estimate the expression level of mRNAs after correction for sequencing depth and transcript length. Differentially expressed mRNAs (DEmRNAs) were defined with a cutoff of log_2_FC > 1 and FDR < 0.05. The heat map based on Z-score was drawn for the DEmRNAs. Gene ontology (GO) annotation and functional enrichment analyses of DEmRNAs were performed using an online database.^1^

### Long Non-coding RNA Identification and Analysis

After reconstruction, we retained the transcripts with length ≥200 bp and exon number ≥1. To improve the reliability in identifying true lncRNAs, we selected the transcripts with no coding potential predicted by both coding potential calculator 2 (CPC2) and coding-non-coding index (CNCI) for further analysis (Sun et al., 2013; Kang et al., 2017). According to the positions of the lncRNAs relative to the protein-coding genes in the genome, we divided the lncRNAs into 5 types, including sense lncRNAs, antisense lncRNAs, intronic lncRNAs, bidirectional lncRNAs, and intergenic lncRNAs. Their genomic distributions were shown by Circos plots using an online platform.^2^ The FPKM values of the lncRNAs were calculated, and DESeq2 was used for the DE analysis (Love et al., 2014). The DElncRNAs were selected with the criteria of log_2_FC >1 and FDR < 0.05. We used RNAplex to predict the complementary relationship between antisense lncRNAs and mRNAs (Tafer and Hofacker, 2008). A prediction of *cis*-acting interactions was made if the location of the lncRNA was upstream or downstream of a gene within 10 kb. On the other hand, a prediction of *trans*-acting interactions was made based on Pearson’s correlation coefficients between lncRNAs and protein-coding genes. Genes with an absolute correlation greater than 0.95 was considered the *tran*s-acting target genes. Pathway enrichment analysis of these genes was performed using the Kyoto Encyclopedia of Genes and Genomes (KEGG) (Kanehisa et al., 2008).

### Circular RNA Identification and Analysis

For circRNA identification, we extracted the unmapped reads from the genome alignment. Afterward, the reads (about 20 bp) at both terminals of these unmapped reads were aligned again to the reference genome using bowtie2 and the mapped reads were referred to as anchor reads (Langmead and Salzberg, 2012). All mapped anchor reads in 6 samples were submitted to the find_circ module in bowtie2 to identify circRNAs (Memczak et al., 2013). Expression levels of circRNAs were calculated by back-spliced reads per million mapped reads (RPM). GO annotation and functional enrichment analysis of the identified circRNAs were performed using an online database (see text footnote 1).

### MicroRNA Identification and Analysis

The total RNA was subjected to agarose gel electrophoresis and small RNAs with 18–30 nucleotides were extracted. Afterward, 3′ and 5′ adapters were added to the small RNAs and reverse transcription and PCR amplification were performed. We extracted and purified the PCR products with a length of 140–160 bp to construct the cDNA library for miRNA and Illumina HiSeq™ 4000 was used for the sequencing. To obtain the clean tags (sequences of miRNA), we filtered low-quality reads (reads containing more than one base with quality value less than 20 or reads containing N), reads with 5′ adapters, reads with long polyA tails (more than 70% of the bases in reads were A), and reads without 3′ adapters. All the clean tags were aligned in GenBank and Rfam databases to remove rRNAs, scRNAs, snoRNAs, snRNAs, and tRNAs. The screened clean tags were searched against the miRBase database to identify known miRNAs in *Medicago truncatula*. Afterward, the unannotated tags were aligned to the reference genome and predicted hairpin structures to identify novel miRNAs. The expression of the miRNAs was represented as TPM (tags per million). DEmiRNAs were identified using edgeR, with dispersion set to 0.01 and other parameters set to default (Robinson et al., 2010).

### Construction and Analysis of the Competing Endogenous RNAs Regulatory Network

Based on the ceRNA theory, we predicted the interaction pairs, DEmiRNAs-DEmRNAs, DEmiRNAs-DElncRNAs, and DEmiRNAs-DEcircRNAs, using Patmatch_v1.2.^3^ Pairwise correlations were calculated using the Spearman correlation coefficient (SCC) and the interaction networks were built using gene pairs with SCC < –0.5. Cytoscape software was used to display the visual models (Shannon et al., 2003).

### Quantitative Real-Time Reverse Transcription-Polymerase Chain Reaction Validation

To validate the accuracy of the whole-transcriptome RNA sequencing, we performed quantitative real-time reverse transcription-polymerase chain reaction (qRT-PCR) for some randomly selected DEmRNAs. *MtActin* served as the internal reference gene. The primers were designed using the Primer 5.0 software and are listed in Supplementary Table 1. First-strand cDNA was synthesized from 1 μg total RNA using TransScript-Uni One-Step gDNA Removal and cDNA Synthesis SuperMix (TransGen Biotech, Beijing, China). The SYBR Premix Ex Taq (Takara, Japan) was used for quantitative detection on the 7,500 Real-Time PCR System (Applied Biosystems, CA, United States). A 20-μl PCR system containing 10 μl 2 × SYBR Premix Ex Taq (Takara, Japan), 8 μl ddH_2_O, 0.8 μl cDNA, 0.4 μl Dye II, and 0.8 μl primers, was used. The qPCR process included the holding stage (95°C for 30 s), cycling stage (95°C for 5 s and 60°C for 34 s), and melt curve stage (95°C for 15 s, 60°C for 1 min, 95°C for 30 s, and 60°C for 15 s). All samples analyses were repeated thrice and the relative expressions of DEmRNAs were calculated using the 2^–Δ^ ^Δ^ *^Ct^* method (Livak and Schmittgen, 2001).

## Results

### Analysis of mRNAs Characteristics in Response to Salt Stress

**FIGURE 1:**
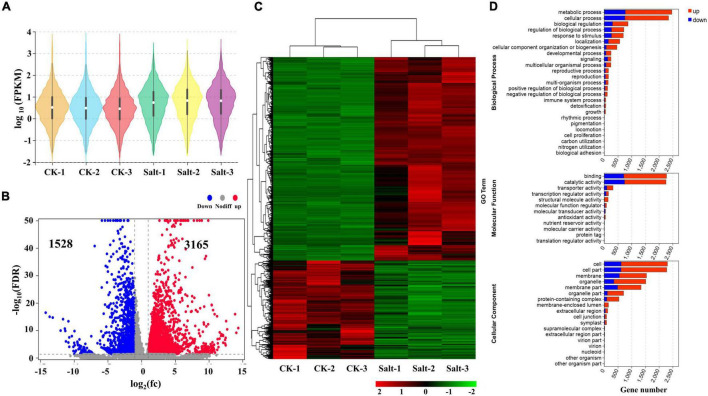
Identification and analysis of mRNAs and DEmRNAs in *Medicago truncatula* leaf under salt stress. **(A)** FPKM distribution of mRNAs in six samples. **(B)** DEmRNAs statistics after salt treatment. **(C)** Heat map of all DEmRNAs. **(D)** GO enrichment of all DEmRNAs.

From the whole-transcriptome RNA sequencing data, we obtained the FPKM values of protein-coding genes. According to the distribution of the expression abundance of the transcripts from the 6 different samples, we found that the overall gene expression levels could be distinctly divided into two groups: the control group treated with water (CK-1, CK-2, and CK-3) and the experimental group treated with salt (Salt-1, Salt-2, and Salt-3) (Figure 1A). This result indicated that the salt treatment used in this study was effective. We identified a total of 4,693 (3,165 up-regulated and 1,528 down-regulated) DEmRNAs (Figure 1B and Supplementary Table 2). A heat map presented the expression profiles of the DEmRNAs and showed that three repeats of each treatment clustered together, while the Salt-treated group and the CK group were clustered separately (Figure 1C). Cluster analysis also revealed that most genes were up-regulated after the salt treatment, while a relatively few genes were down-regulated (Figure 1C). To explore the functions of the DEmRNAs, we performed a GO enrichment analysis. Based on the GO enrichment analysis, we found that, in terms of biological process, most of the DEmRNAs were annotated to metabolic and cellular processes, and in terms of molecular function, the two largest categories were annotated to binding and catalytic activities. In addition, two major terms (cell and cell part of cellular component) were also enriched (Figure 1D and Supplementary Table 3). These enrichment analyses suggested that these GO terms might be involved in the salt stress response of *Medicago truncatula*. KEGG pathway analysis showed that the metabolic pathways were the most enriched (Supplementary Figure 1).

**FIGURE 2:**
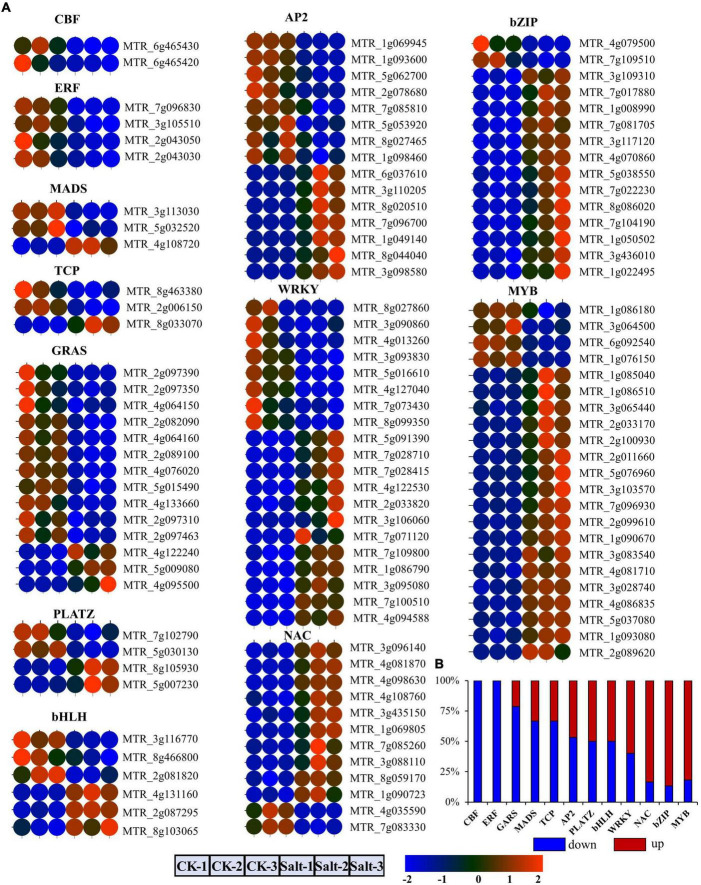
Identification and analysis of differentially expressed TFs under salt stress. **(A)** The expression level of 12 TFs in six samples. **(B)** Histogram of up-regulated and down-regulated genes in 12 TF families.

Subsequently, we identified 12 transcription factor families from the DEmRNAs (Figure 2). By comparing the expression levels of the transcription factors (TFs) among the 6 different samples, we divided them into 4 groups. First, the core-binding factor (CBF) and ethylene-responsive factor (ERF) TF families, including 2 and 4 differentially expressed TFs, respectively, were all down-regulated after salt stress (Figure 2A). Second, more than 50% of the DEmRNAs belonging to the GRAS, MADS, and TCP TF families were down-regulated (Figure 2A). Third, 50% of the DEmRNAs in the bHLH, PLATA, WRKY, and AP2 transcription factor families were down-regulated under salt stress (Figure 2B). Fourth, most differentially expressed TFs in the MYB, bZIP, and NAC TF families were up-regulated in response to salt stress (Figure 2B).

### Analysis of Long Non-coding RNAs Characteristics in Response to Salt Stress

**FIGURE 3:**
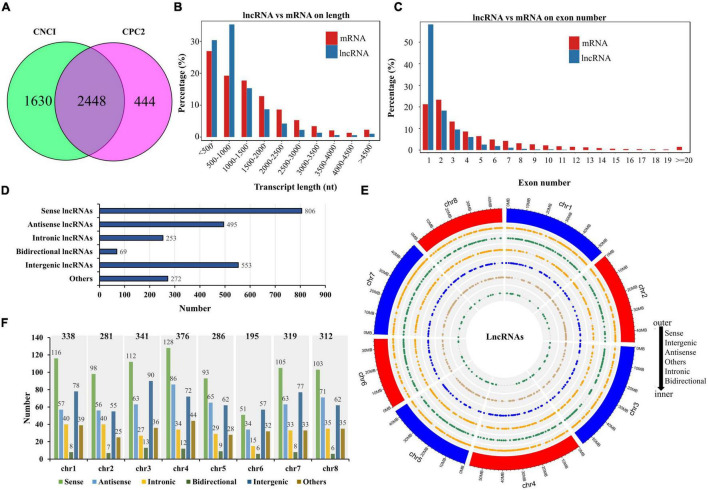
Identification and distribution of lncRNAs. **(A)** The number of lncRNAs identified by the CNCI and CPC2 methods. **(B)** Comparison of lncRNA with mRNA in terms of transcript length. **(C)** Comparison of lncRNA with mRNA in terms of exon number. **(D)** Number statistics of different lncRNAs types. **(E)** Genomic distribution of different lncRNAs types. **(F)** Number statistics of different lncRNAs types on eight chromosomes.

To improve the accuracy of lncRNA identification before salt stress, we used the CNCI and CPC2 software to predict the coding potential of transcripts and selected the common transcripts without coding potential as reliable lncRNAs. Therefore, we obtained a total of 2,448 lncRNAs (Figure 3A and Supplementary Table 4). Comparing the length between the lncRNA and mRNA, we found that their length distributions were similar (most transcripts were less than 1,500 nucleotides), except that ratio of lncRNAs with 500–1,000 nucleotides was higher than that of mRNAs with the same nucleotide length (Figure 3B). The exon number in the mRNAs was more widely distributed than that of the lncRNAs, mostly ranging from 1 to 4, and the percentage of the lncRNAs with one exon was much higher than that of the mRNAs (Figure 3C). According to the position of the lncRNAs relative to the protein-coding genes in the genome, we identified 806 sense lncRNAs, 495 antisense lncRNAs, 253 intronic lncRNAs, 69 bidirectional lncRNAs, 553 intergenic lncRNAs, and 272 other lncRNAs (Figure 3D). The genomic location and number distribution on 8 chromosomes of these different types of lncRNAs are shown in Figures 3E,F, respectively.

**FIGURE 4:**
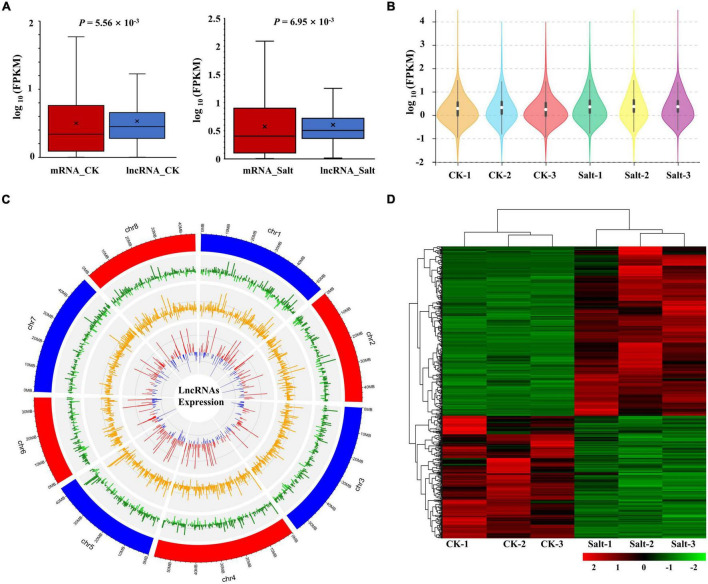
Expression analysis of lncRNAs. **(A)** Comparison of the expression levels between lncRNAs and mRNAs in the CK and Salt groups. **(B)** FPKM distribution of lncRNAs in six samples. **(C)** Genomic distribution of lncRNAs. The three circles (from outer to inner) represented the expression levels (log_10_FPKM) of lncRNA in the CK group, lncRNA in the Salt group, and fold change of the expression levels, respectively. **(D)** The heat map of all DElncRNAs.

Subsequently, we performed lncRNA expression analysis and, based on the FPKM values, we found that lncRNA expression was significantly different from that of mRNA either in the CK group or the Salt group (Figure 4A). Compared with the CK group, the overall expression levels of lncRNAs were more similar within the Salt group, which was consistent with those of mRNAs (Figures 1A, 4B). The expression levels of lncRNA in the CK and Salt groups and their fold changes are shown in Figure 4C. We obtained a total of 293 up-regulated lncRNAs and 212 down-regulated lncRNAs (Figures 4C,D and Supplementary Table 5). A heat map of these DElncRNAs showed that three repeats of each treatment clustered together (Figure 4D).

**FIGURE 5:**
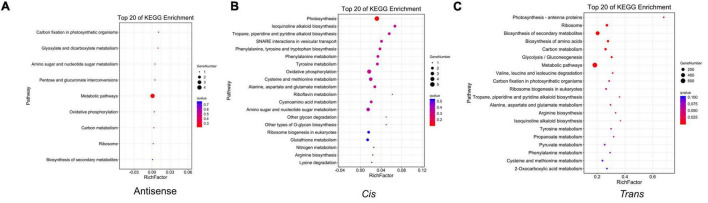
The KEGG pathway analysis of the target genes of DElncRNAs. **(A)** The KEGG pathway analysis of the antisense-targeted genes of DElncRNAs. **(B)** The KEGG pathway analysis of *cis*-targeted genes of DElncRNAs. **(C)** The KEGG pathway analysis of *trans*-targeted genes of DElncRNAs.

According to complementary base pairing, we predicted antisense lncRNAs. The result showed that 496 lncRNAs and 488 mRNAs formed 541 antisense pairs, while between DElncRNAs and DEmRNAs, only 31 pairs were identified (Table 1 and Supplementary Table 6). Afterward, the KEGG pathway analysis was performed on the antisense-targeted DEmRNAs and metabolic pathways were enriched with the most gene numbers (Figure 5A). We used a 10-kb window upstream or downstream of the lncRNA to identify *cis-*interactions and identified 191 DElncRNA-DEmRNA pairs (Table 1 and Supplementary Table 7). The *cis*-targeted DEmRNAs were enriched in photosynthetic pathways with a minimum *q*-value (Figure 5B). Correlation analysis revealed 419,734 *trans*-interactions between DElncRNAs and DEmRNAs (Table 1). KEGG pathway analysis showed that the metabolic pathway and biosynthesis of secondary metabolites pathway were the most enriched (Figure 5C). The results of the GO enrichment analysis of antisense, *cis*, and *trans* lncRNAs are shown in Supplementary Figure 2.

**TABLE 1:** Association analysis between lncRNA and mRNA.

Association	List	lncRNA	mRNA	Pair
Antisense	All	496	488	541
	Diff	31	30	31
*Cis*	All	2,328	5,554	6,842
	Diff	160	168	191
*Trans*	All	2,448	48,179	1.18 E^08^
	Diff	505	4,675	419,734

### Analysis of Circular RNAs Characteristics in Response to Salt Stress

**FIGURE 6:**
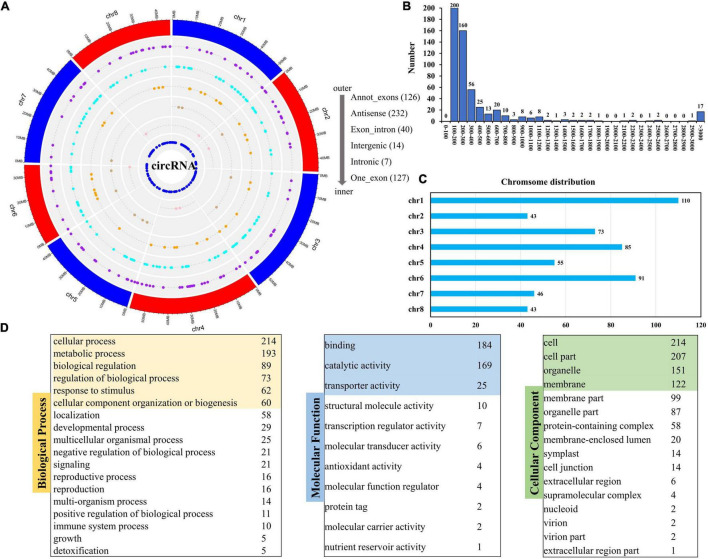
Distribution of circRNAs and GO analysis of source genes. **(A)** Genomic distribution of different circRNAs types. **(B)** Number statistics of different circRNAs in length. **(C)** Chromosome distribution of circRNAs. **(D)** GO enrichment of all source genes of circRNAs.

In this study, we identified a total of 546 circRNAs that could be classified into 6 types (Figure 6A and Supplementary Table 8). The genomic distribution of the different types of circRNAs is presented in Figure 6A. In terms of length, most circRNAs were shorter than 400 bp (Figure 6B). Different chromosomes included different numbers of circRNAs. Chromosome 1 (Chr. 1) had the most circRNAs (Figure 6C). GO annotation of the circRNA genes showed that, in terms of biological process, they were enriched in cellular process, metabolic process, and biological regulation; in terms of molecular function, there were enriched in binding, catalytic, and transport activities; whereas, in terms of cellular component, they were enriched in cell, cell part, and organelle (Figure 6D).

**FIGURE 7:**
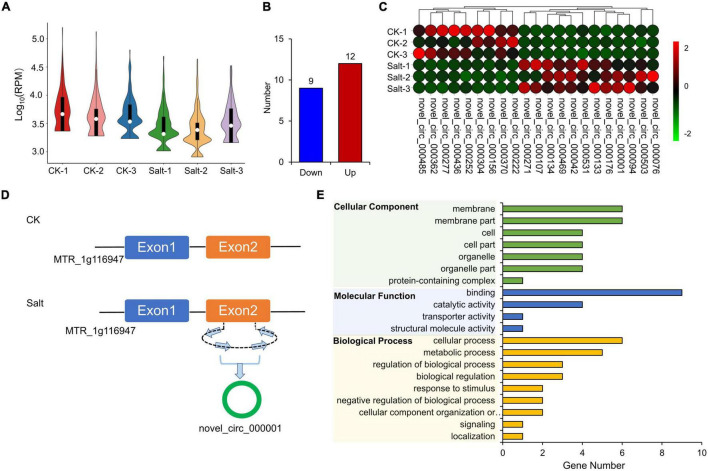
Expression analysis of circRNAs. **(A)** FPKM distribution of cirsRNAs in six samples. **(B)** Statistics of down-regulated and up-regulated DEcircRNAs. **(C)** Expression visualization of DEcircRNAs in six samples. **(D)** MTR_1g116947 formed novel_circ_000001. **(E)** GO enrichment of source genes of DEcircRNAs.

The expression levels of circRNAs in the 6 samples were visualized in a violin plot (Figure 7A). We identified a total of 21 DEcircRNAs (12 up-regulated and 9 down-regulated, Figure 7B and Supplementary Table 9). Their IDs and expressions in six samples are shown in Figure 7C and Supplementary Table 9, respectively. Novel_circ_000001 is one of the DEcircRNAs and MTR_1g116947 is its source gene. In the CK group, novel_circ_000001 was not expressed. However, after salt treatment, the second exon of MTR_1g116947 formed a circRNA, which resulted in a significantly higher expression of novel_circ_000001 (Figure 7D). We performed the GO enrichment analysis on all the source genes of DEcircRNAs and found that membranes under the cellular components, binding activities under the molecular functions, and cellular processes under the biological process contained the largest number of source genes (Figure 7E).

### Analysis of MicroRNAs Characteristics in Response to Salt Stress

**FIGURE 8:**
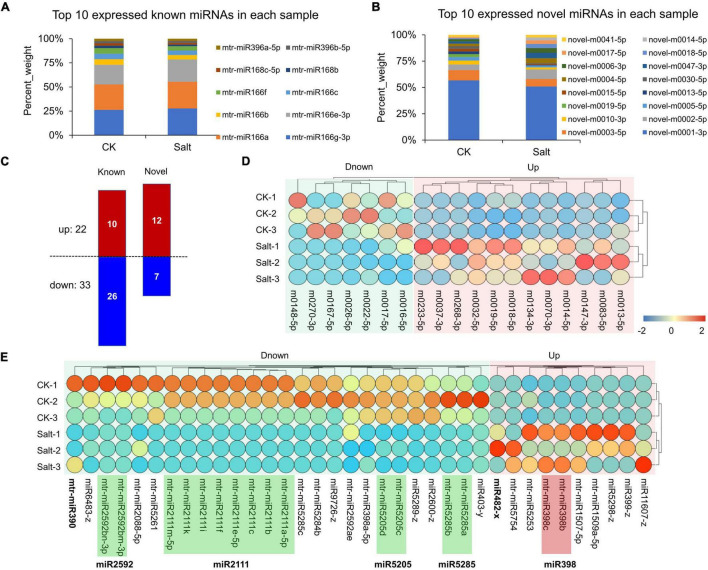
Expression analysis of miRNAs. **(A)** Top 10 expressions of known miRNAs in the CK and Salt groups. **(B)** Top 10 expressions of novel miRNAs in the CK and Salt groups. **(C)** Statistics of down-regulated and up-regulated DEmiRNAs. **(D)** Expression visualization of novel DEmiRNAs in six samples. **(E)** Expression visualization of known DEmiRNAs in six samples.

Through whole-transcriptome RNA sequencing and reads alignment, we identified multiple known and novel miRNAs (Supplementary Table 10). The top 10 known and novel miRNAs in the CK and Salt groups are shown in Figures 8A,B, respectively. Of note, mtr-miR166 and novel-m0001-3p were most highly expressed. For novel miRNAs, 19 DEmiRNAs (12 up-regulated and 7 down-regulated) were identified (Figure 8C). Their expression levels in the 6 samples (3 for both the CK and Salt groups) are shown in Figure 8D. For the known miRNAs, 36 DEmiRNAs (10 up-regulated and 26 down-regulated) were identified (Figure 8C). For these miRNAs, 8 miRNAs belonging to the miR2111 family, 2 miRNAs belonging to the miR2592 family, 2 miRNAs belonging to the miR5205 family, and 2 miRNAs belonging to the miR5285 family were down-regulated; in contrast, 2 miRNAs belonging to the miR398 family were up-regulated (Figure 8E).

### Construction of the Competing Endogenous RNA Regulatory Network in Response to Salt Stress

**FIGURE 9:**
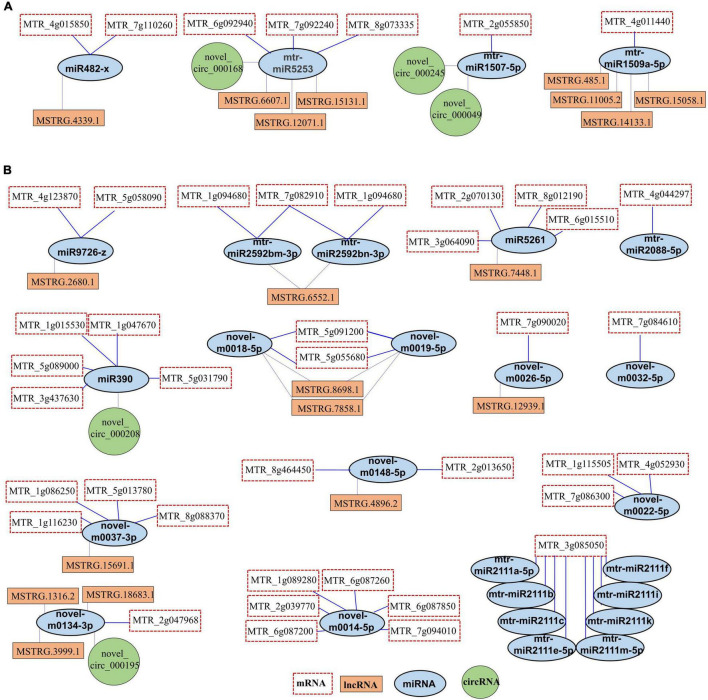
The ceRNA networks. **(A)** The ceRNA networks were constructed with up-regulated miRNAs. **(B)** The ceRNA networks were constructed with down-regulated miRNAs.

To explore the relationship between protein-coding RNAs and ncRNAs under salt stress, we constructed the ceRNA regulatory networks based on the ceRNA theory. Between DEmiRNAs and mRNAs, we found a total of 524 pairs of interactions (Supplementary Table 11). There were 10 pairs between DEmiRNAs and DEcircRNAs and 46 pairs between DEmiRNAs and lncRNAs (Supplementary Tables 13, 14). A total of 42 DEmRNAs were predicted as targets of 27 DEmiRNAs (Figure 9 and Supplementary Table 12). Moreover, 19 lncRNAs and 5 DEcircRNAs were predicted as DEmiRNA sponges in salt stress response in leaves of *Medicago truncatula* (Figure 9). In the up-regulated miRNAs, we found 4 DEmiRNAs formed 7 DEmiRNA-DEmRNA, 8 DEmiRNA-lncRNA, and 3 DEmiRNA-DEcircRNA interactions (Figure 9A). Most of the down-regulated miRNAs were involved in multiple pairs with DEmRNAs and DEceRNAs (Figure 9B). Novel-m0018-5p and novel-m0019-5p interacted with the same DEmRNAs and lncRNAs. The mtr-miR2111 family included 8 DEmiRNAs, targeted MTR_3g085050 (Figure 9B). These DEceRNA, DEmiRNA, and DEmRNA might act as important regulators for salt stress response in *Medicago truncatula*.

### Analysis of Key Pathways in Response to Salt Stress

**FIGURE 10:**
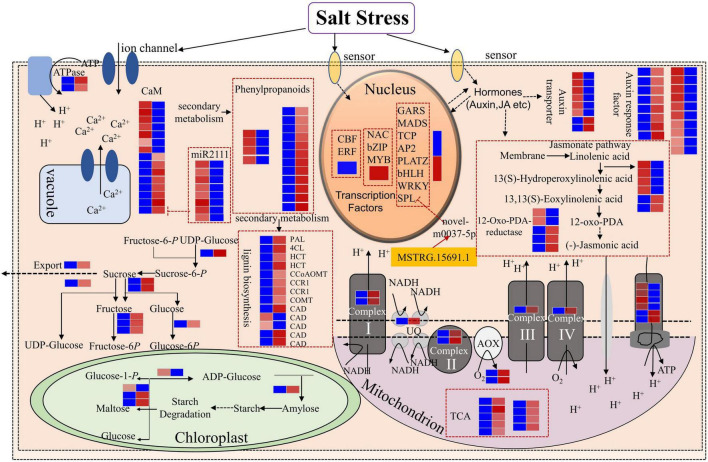
The important pathways of *Medicago truncatula*’s response to salt stress and the relationship between DEmRNAs and ceRNAs. Blue boxes represent lower expression lever and red boxes represent higher expression level.

According to the results of whole-transcriptome RNA sequencing, we analyzed genes and pathways that are important in response to salt stress in *Medicago truncatula*. In the nucleus, TFs act as important regulators for gene expression. In this study, we identified TFs, including 2 CBFs (MTR_6g465430 and MRT-6g465420) and 4 ERFs (MTR_7g096830, MTR_3g105510, MTR_2g043050, and MTR_2g043030), were down-regulated after salt treatment, whereas most genes in the NAC, bZIP, and MYB family were up-regulated (Figures 2, 10). SPL transcription factors were negatively regulated by novel-m0037-5p and novel-m0037-5p interaction with the lncRNA MSTRG.15691.1 (Figures 9B, 10). These results suggested that TFs themselves or TFs in relationship with non-coding RNAs play important roles in salt stress response in *Medicago truncatula*.

In this study, we found that 8 genes related to calmodulin (CaM) were up-regulated, including MTR_3g085050, which was negatively regulated by miR2111. The 8 members belonging to the miR2111 family were down-regulated, thus resulting in the up-regulation of MTR_3g085050, which encodes a calmodulin-binding protein (Figures 9B, 10 and Supplementary Table 15). We simultaneously identified 7 down-regulated *CaM* genes. The significant changes in the expression levels of the *CaM* genes indicated that Ca^+^ signal transduction was essential for salt stress response in *Medicago truncatula* and that different *CaM* genes functioned differently to regulate Ca^+^ concentration balance and downstream signals.

Genes involved in carbohydrate metabolism in the chloroplast also responded to salt stress (Figure 10). 1 gene (MTR_4g131760) related to starch synthesis (in the first step) was down-regulated and 2 genes (MTR_1g019440 and MTR_2g020240) related to starch degradation were up-regulated. In addition, eight differentially expressed genes related to sucrose metabolism were up-regulated (Figure 10 and Supplementary Table 15).

In the plant hormones pathway, 6 genes related to auxin transport were down-regulated, while 2 genes were up-regulated after salt treatment. In addition, 8 auxin response factors were up-regulated, while 10 related factors were down-regulated (Figure 10 and Supplementary Table 15). In the jasmonate pathway, 4 genes participating in the process from linolenic acid to hydroperoxylinolenic acid were down-regulated, while 2 genes were up-regulated. 2 12-oxo-PDA-reductases genes were down-regulated, while 3 genes were up-regulated (Figure 10 and Supplementary Table 15).

In the phenylpropanoid metabolism, we found that 12 genes related to phenylpropanoid biosynthesis were up-regulated, while only 4 genes were down-regulated (Figure 10 and Supplementary Table 15). In the secondary metabolism, only 2 genes (MTR_5g031300 and MTR_5g031360) related to cinnamyl-alcohol dehydrogenase (CAD) were down-regulated, while other 11 genes related to lignin biosynthesis were all up-regulated (Figure 10 and Supplementary Table 15). Further analyses of the lignin content are necessary to explore the relationship between lignin genes and salt tolerance in *Medicago truncatula*.

In the mitochondrion, genes related to 4 electron transport protein complexes, tricarboxylic acid cycle (TCA), and alternative oxidase (AOX) were up-regulated (Figure 10 and Supplementary Table 15). This revealed that the salt stress affected the reactions in the mitochondrion.

Based on the candidate genes involved in the key pathways implicated in response to salt stress, we further analyzed their regulatory relationship with lncRNAs. The result revealed that 6 and 81 genes may be *cis-* and *trans-*regulated by lncRNAs, respectively (Supplementary Table 15). This suggested that complex mechanisms, including interactions between mRNAs and lncRNAs, might respond to salt stress and that the fine control module worth further study.

### Validation of the Expression of RNA by Quantitative Real-Time Reverse Transcription-Polymerase Chain Reaction

To confirm the results of the whole-transcriptome RNA sequencing, we used qRT-PCR to verify the expressions of randomly selected DERNAs, DEmiRNAs and lncRNAs. As shown in Supplementary Figure 3, the qRT-PCR results were consistent with the RNA-seq data. MTR_8g059170 was up-regulated, while the other 5 genes were significantly down-regulated after the salt treatment. And, we also found the miRNAs and its target genes had an opposite expression patterns (mtr-miR2088-5p and MTR_4g044297, novel-m0148-5p, and MTR_8g464450). These results demonstrate the accuracy of the RNA-seq data in this study.

## Discussion

Salt stress is among the most severe abiotic stresses that threaten plant growth (Butcher et al., 2016). With the expansion of saline land, there is a need to broaden the current knowledge on plant responses to salt stress. To date, an immense amount of research has been performed to assess different plants’ responses to salinity; however, there are still many unknown regulatory elements and processes, including transcription and post-transcriptional regulation. In this study, we identified multiple key salt stress response factors through whole-transcriptome sequencing on the leaves of *Medicago truncatula*, a legume model crop, by comparing salt and water treated groups.

Based on the results of the whole-transcriptome sequencing, we first analyzed the protein-coding mRNA characteristics in response to salt stress in *Medicago truncatula* leaves. It has been recognized that many TFs have an important role in stress-responsive transcription, such as bZIP, AP2/ERF, MYB, NAC, and WAKY (Golldack et al., 2011). Yang et al. (2009) reported that *AtbZIP24* was induced by salt stress in *Arabidopsis thaliana* but suppressed in the salt-tolerant relative *Lobularia maritima*. Liu et al. (2007) reported that salt stress in *Arabidopsis thaliana* induced a signaling cascade involving the processing of *AtbZIP17*. Among the differentially expressed TFs in this study, 15 bZIP TFs (13 were highly induced) were induced by salt stress in *Medicago truncatula* leaves and their functions worth further study. We also found that 4 ERFs were down-regulated after salt treatment, suggesting that ERFs may be involved in the salt response. Cheng et al. (2013) reported that *ERF1* was highly induced by high salinity in *Arabidopsis* and that *ERF1*-overexpressing *Arabidopsis* lines were more tolerant to salt stress. It has been demonstrated that the overexpression of *WAKY25* and *WAKY33* could improve salt tolerance in *Arabidopsis* and that other WRKY-type TFs involved in salt response were also supported in rice and halotolerant grass *Festuca rubra* ssp. *litoralis* (Diédhiou et al., 2009a,b; Jiang and Deyholos, 2009). We identified 20 differentially expressed WRKY TFs; however, there was no consistent expression change in them, which may due to different functions in response to saline stress. Moreover, members in the MYB and bHLH families were also reported in response to ABA and ROS signaling related to salt adaptation (Lippold et al., 2009; Golldack et al., 2011). Our study also found some differentially expressed MYB and bHLH TFs. These results suggest that the differentially expressed TFs identified in *Medicago truncatula* might be involved in the complex regulatory systems in salt response as seen in other plants. Also, our results provide potential opportunities for improving salt tolerance in *Medicago truncatula* and *Medicago sativa*.

Recently, non-coding RNAs, including lncRNAs, circRNAs, and miRNAs, have been shown to play crucial regulatory roles in diverse biological processes involving complex mechanisms. Wang et al. (2015) identified the lncRNAs involved in salt stress in *Medicago truncatula* and predicted the interaction networks among the lncRNAs and protein-coding RNAs. In this study, a total of 2,448 lncRNAs were identified, which is quite different from the previous identification results (Wang et al., 2015). This phenomenon may be caused by different analysis methods in different studies. Zhang et al. (2021) identified lncRNA354 and found that its expression was reduced in salt-treated cotton. Silencing lncRNA354 enhanced the resistance to salt stress in cotton. The lncRNA DRIR could be significantly activated by drought and salt stress; moreover, its overexpression in *Arabidopsis* increased tolerance to salt stress (Qin et al., 2017). For circRNAs, Li et al. (2021) analyzed their expression patterns and functions between salt-sensitive and salt-tolerant poplars and concluded that circRNAs might regulate the gene expression of woody poplars efficiently in the salt tolerance of different poplars. Based on a previous transcriptome-wide analysis of circRNAs in rice (Lu et al., 2015), Zhou et al. (2021) used a multiplexed CRISPR-Cas9 strategy to efficiently acquire individual null mutants for 4 circRNAs (Os02circ25329, Os06circ02797, Os03circ00204, and Os05circ02465) in rice and revealed that they all participated in salt stress response during seed germination. Furthermore, through molecular and computational analyses, a previous study demonstrated that Os06circ02797 could bind and sequester OsMIR408. Long et al. (2015) analyzed salt-stress-regulated miRNAs from roots of *Medicago* and found that different expression levels of some miRNAs were perhaps a consequence of the long-term adaptive evolution. In maize, miR169q was found to respond to stress-induced ROS signals and negatively regulate seedling salt tolerance. MiR169q repressed the transcript abundance of its target *NUCLEAR FACTOR YA8* (*ZmNF-YA8*), whose high expression improved salt tolerance in maize (Xing et al., 2021). In poplar, He et al. (2018) found that miR390 overexpression stimulated lateral root development and increased salt tolerance. The miR390/ARFs (auxin response factors) module is a key regulator subjected to salt stress by modulating the auxin pathway. In this study, we found that miRNA390 was significantly down-regulated in *Medicago truncatula* leaves after salt stress (Figure 8E). We also identified some ARFs, whose expression levels changed significantly (Figure 10). These results suggest that the miR390/ARFs module may play a role in *Medicago truncatula* leaves in response to salinity stress. MiRNA482 was reported to be involved in immune and drought response in plants (Tang and Chu, 2017; Song et al., 2019; Waititu et al., 2020). In this study, we found that miRNA482 was induced by salt stress (Figure 8E). This discovery may broaden the current knowledge on the function of miRNA482. MiR398 is directly related to the plant stress regulation network, including those regulating plants’ responses to salt (Tang and Chu, 2017; Song et al., 2019; Chen et al., 2020; Waititu et al., 2020), which was consistent with our results (Figure 8E). The miR2111, which was reported to be related to legume susceptibility to rhizobial infection and root competence for nodulation (Tsikou et al., 2018), was found to be down-regulated under salt stress in *Medicago truncatula*. Compared with other plant studies, we also identified many new RNAs. This difference might be due to the fact that different studies used different salt concentrations at different periods to simulate salt stress.

## Conclusion

In summary, it is known that protein-coding and non-coding RNAs and their interactions are essential for plant response to salt stress. However, the molecular mechanisms underlying the ceRNA network remain unknown. Our study comprehensively analyzed the coding and non-coding RNAs in *Medicago truncatula* after salt or water treatments and showed the interactions between them are important for regulating the salt stress response. Furthermore, we identified the DERNAs as salt response factors and displayed the intracellular pathways implicated in response to salt stress. Our results provide helpful information for further molecular function studies and breeding practices in *Medicago*.

## Data Availability Statement

The datasets presented in this study can be found in online repositories. The names of the repository/repositories and accession number(s) can be found below: National Center for Biotechnology Information (NCBI) BioProject database under accession number PRJNA813502.

## Author Contributions

YA: samples collection, data analysis, and original draft preparation. HS: figures and tables preparation. QN: data validation and analysis. SY: manuscript review and editing. All authors contributed to the article and approved the submitted version.

## Conflict of Interest

The authors declare that the research was conducted in the absence of any commercial or financial relationships that could be construed as a potential conflict of interest.

## Publisher’s Note

All claims expressed in this article are solely those of the authors and do not necessarily represent those of their affiliated organizations, or those of the publisher, the editors and the reviewers. Any product that may be evaluated in this article, or claim that may be made by its manufacturer, is not guaranteed or endorsed by the publisher.

## Footnotes

1
http://www.geneontology.org/

2
https://yimingyu.shinyapps.io/shinycircos/

3
https://www.arabidopsis.org/cgi-bin/patmatch/nph-patmatch.pl

## Supplementary Material

Supplementary Figure 1 The KEGG pathway analysis of DEmRNAs.

Supplementary Figure 2 GO enrichment of the target genes of antisense, *cis*-acting, and *trans*-acting DElncRNAs. Green: Molecular Function; Purple: Cellular Component; blue: Biological Process.

Supplementary Figure 3 qRT-PCR validation of the expression levels of RNAsin the CK and Salt groups.

Supplementary Table 1 The primers used in qRT-PCR validation.

Supplementary Table 2 The list of DEmRNAs.

Supplementary Table 3 GO enrichment of DEmRNAs.

Supplementary Table 4 The list of lncRNAs.

Supplementary Table 5 The list of DElncRNAs.

Supplementary Table 6 The list of antisense pairs between DElncRNAs and DEmRNAs.

Supplementary Table 7 The list of *cis*-acting between DElncRNAs and DEmRNAs.

Supplementary Table 8 The list of circRNAs.

Supplementary Table 9 The list of DEcircRNAs.

Supplementary Table 10 The list of DEmiRNAs.

Supplementary Table 11 The list of DEmiRNA-mRNA interactions.

Supplementary Table 12 The list of DEmiRNA-DEmRNA interactions.

Supplementary Table 13 The list of DEmiRNA-DEcircRNA interactions.

Supplementary Table 14 The list of DEmiRNA-lncRNA interactions.

Supplementary Table 15 The list of differentially expressed genes involved in important pathways implicated in response to salt stress in *Medicago truncatula*.

## Abbreviations

DEmRNA: differentially expressed mRNA
lncRNA: long non-coding RNA
circRNA: circular RNA
miRNA: microRNA
FPKM: fragments per kilobase per million reads
RPM: back-spliced reads per million mapped reads
FDR: false discovery rate
GO: Gene Ontology
KEGG: Kyoto Encyclopedia of Genes and Genomes
qRT-PCR: quantitative real-time reverse transcription-polymerase chain reaction.
