# Supplementary figures and images for: Integrated Analysis of Coding and Non-coding RNAs Reveals the Molecular Mechanism Underlying Salt Stress Response in Medicago truncatula
Source: Front Plant Sci. 2022 Apr 18;13:891361. doi: 10.3389/fpls.2022.891361 (PMC9064118; doi:10.3389/fpls.2022.891361)

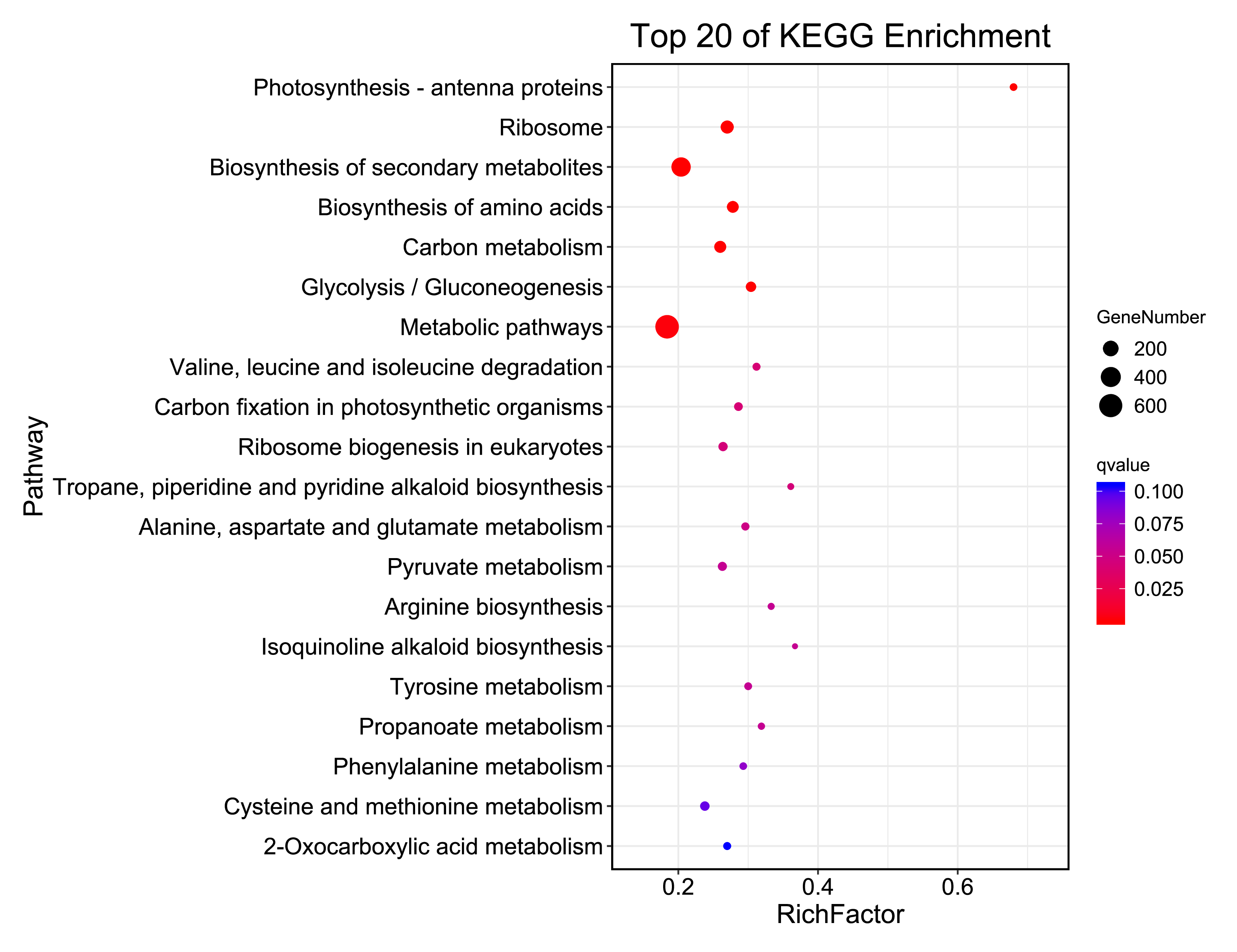

Supplement: Supplementary Figure 1 — The KEGG pathway analysis of DEmRNAs. [file Image_1.TIFF]

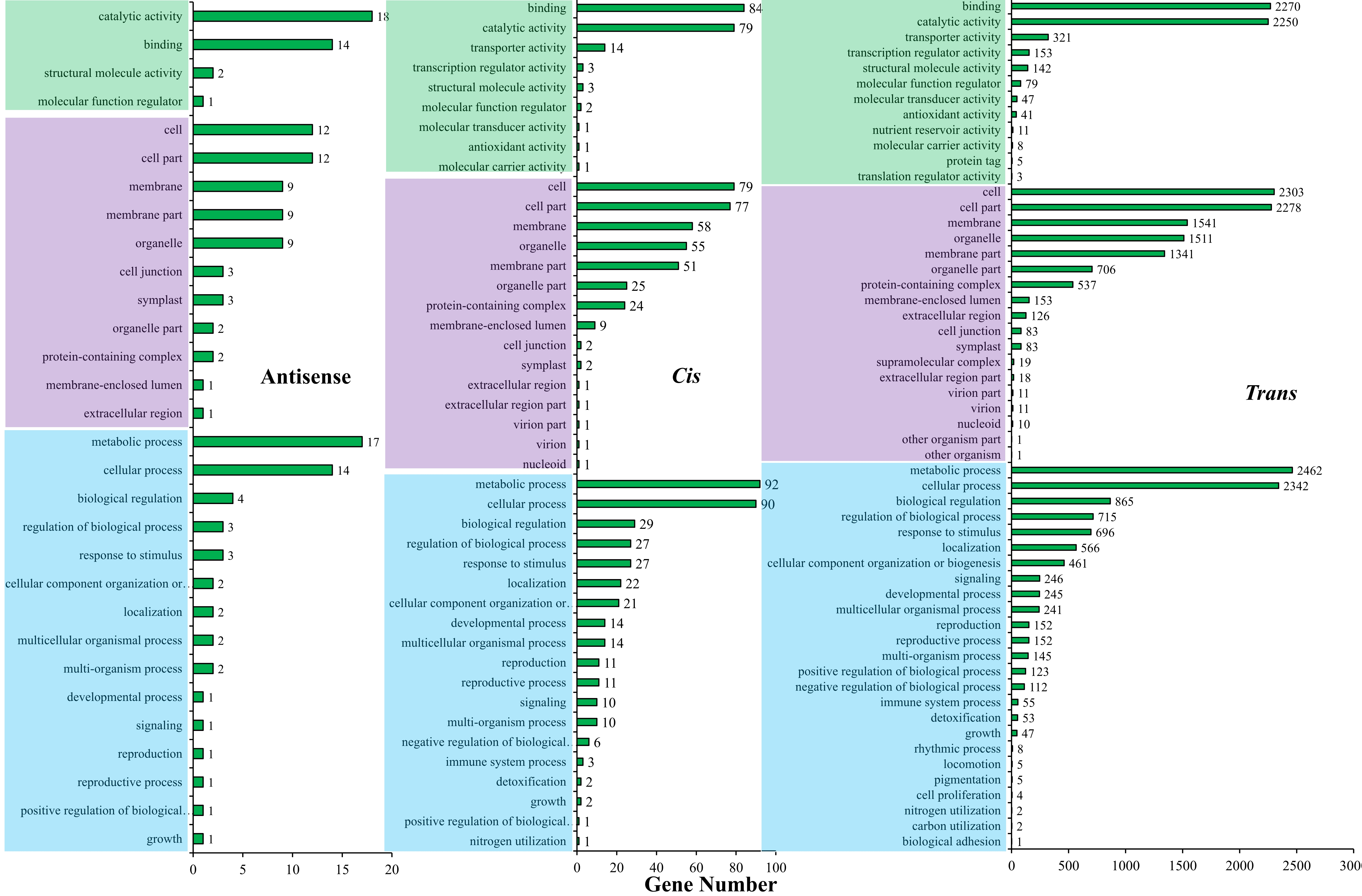

Supplement: Supplementary Figure 2 — GO enrichment of the target genes of antisense, cis-acting, and trans-acting DElncRNAs. Green: Molecular Function; Purple: Cellular Component; blue: Biological Process. [file Image_2.TIFF]

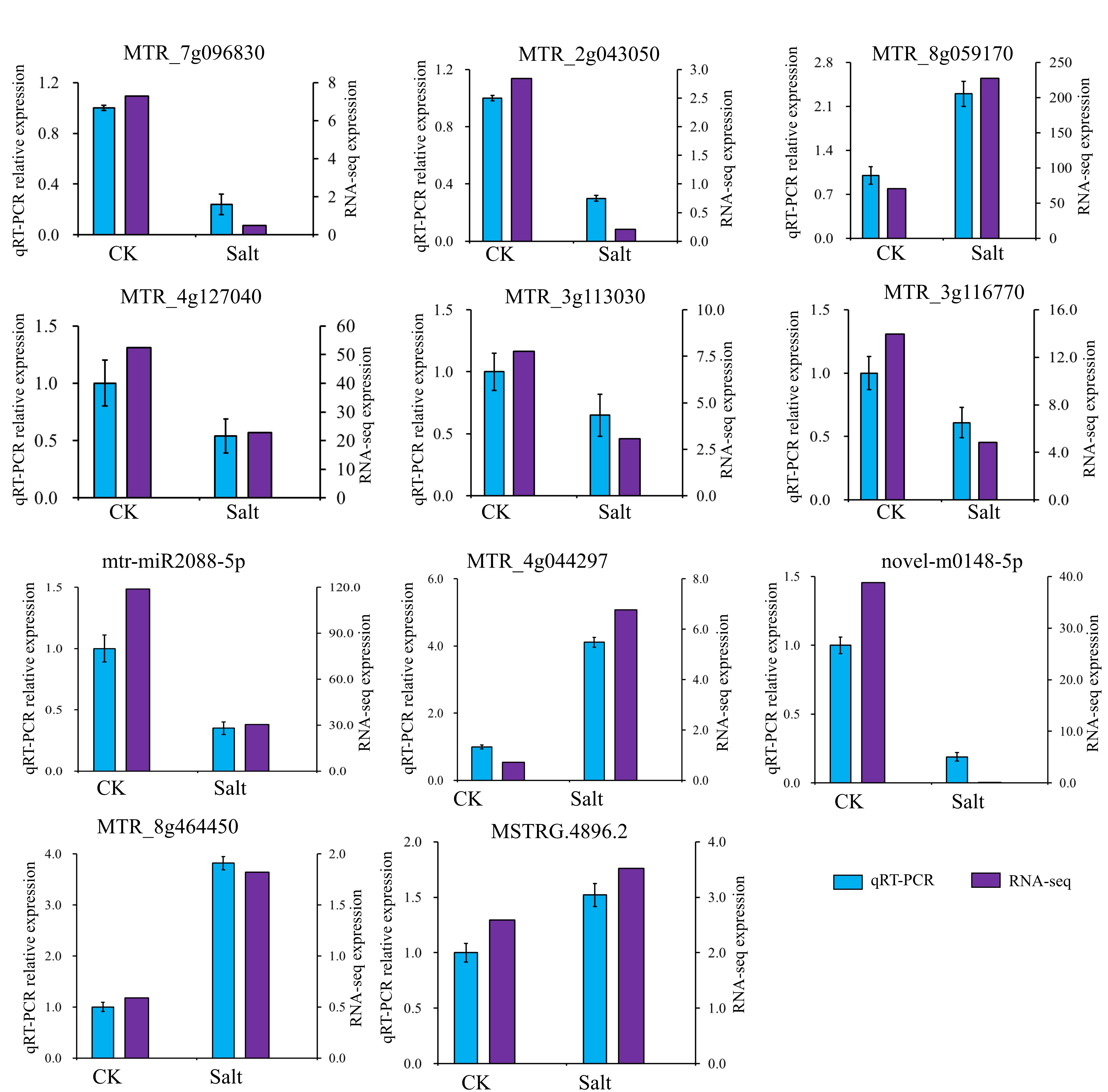

Supplement: Supplementary Figure 3 — qRT-PCR validation of the expression levels of RNAsin the CK and Salt groups. [file Image_3.TIFF]
